# Lung adenocarcinoma-related target gene prediction and drug repositioning

**DOI:** 10.3389/fphar.2022.936758

**Published:** 2022-08-23

**Authors:** Rui Xuan Huang, Damrongrat Siriwanna, William C. Cho, Tsz Kin Wan, Yan Rong Du, Adam N. Bennett, Qian Echo He, Jun Dong Liu, Xiao Tai Huang, Kei Hang Katie Chan

**Affiliations:** ^1^ >Department of Electrical Engineering, City University of Hong Kong, Hong Kong, China; ^2^ >Department of Biomedical Sciences, City University of Hong Kong, Hong Kong, China; ^3^ >Department of Clinical Oncology, Queen Elizabeth Hospital, Hong Kong, China; ^4^ >Department of Linguistics and Modern Languages, The Chinese University of Hong Kong, Hong Kong, China; ^5^ >School of Computer Science and Technology, Xidian University, Xi’an, China; ^6^ >Department of Epidemiology and Center for Global Cardiometabolic Health, Brown University, Providence, RI, United States

**Keywords:** lung adenocarcinoma, drug repositioning, gene prediction, graph attention networks, machine learning, deep learning

## Abstract

Lung cancer is the leading cause of cancer deaths globally, and lung adenocarcinoma (LUAD) is the most common type of lung cancer. Gene dysregulation plays an essential role in the development of LUAD. Drug repositioning based on associations between drug target genes and LUAD target genes are useful to discover potential new drugs for the treatment of LUAD, while also reducing the monetary and time costs of new drug discovery and development. Here, we developed a pipeline based on machine learning to predict potential LUAD-related target genes through established graph attention networks (GATs). We then predicted potential drugs for the treatment of LUAD through gene coincidence-based and gene network distance-based methods. Using data from 535 LUAD tissue samples and 59 precancerous tissue samples from The Cancer Genome Atlas, 48,597 genes were identified and used for the prediction model building of the GAT. The GAT model achieved good predictive performance, with an area under the receiver operating characteristic curve of 0.90. 1,597 potential LUAD-related genes were identified from the GAT model. These LUAD-related genes were then used for drug repositioning. The gene overlap and network distance with the target genes were calculated for 3,070 drugs and 672 preclinical compounds approved by the US Food and Drug Administration. At which, bromoethylamine was predicted as a novel potential preclinical compound for the treatment of LUAD, and cimetidine and benzbromarone were predicted as potential therapeutic drugs for LUAD. The pipeline established in this study presents new approach for developing targeted therapies for LUAD.

## Introduction

Lung cancer is one of the most common cancers globally. In 2020, approximately 2.21 million people had lung cancer, and there were 1.8 million lung cancer-related deaths, which was the highest cancer mortality rate in that year (World Health Organization, 2021). Lung adenocarcinoma (LUAD) belongs to the non-small cell lung cancer (NSCLC) family, which accounts for 40% of all lung cancer types. LUAD is the most common type of lung cancer (Zappa and Mousa, 2016). It has unique cellular and molecular characteristics compared with other lung cancer subtypes. Previous studies have shown that high or low expression levels of certain genes in cancer tissues play a vital role in the development of LUAD (Chen et al., 2019). The dysregulation of these genes provides an opportunity for targeted therapy. Targeted therapy is currently one of the options for the postoperative treatment of patients with LUAD. For example, the epithelial growth factor receptor (EGFR) inhibitors, gefitinib, and erlotinib are already used to treat LUAD patients (Janku et al., 2010; Pao and Chmielecki, 2010). Due to the large number of genes involved in the development of LUAD, the therapeutic effects of inhibiting only a single gene may be limited. Therefore, it is necessary to discover potential genes related to LUAD and identify drugs that target these genes (Cho, 2013).

Targeted therapy refers to a treatment targeting previously defined carcinogenic sites at the cellular and molecular levels. The site may be a protein, a gene, or a gene fragment inside the tumor cell (American Cancer Society, 2021). Targeted therapies interfere with cancer-causing or tumor growth-promoting molecules or genes to treat cancer. The development of new drugs based on cancer-related genes is time-consuming and labor-intensive, and it takes an average of 13 years and $870 million from development to market (Paul et al., 2010; Sertkaya et al., 2016). Therefore, repurposing drugs that have already been approved is a more cost-effective approach. Hundreds of drugs have been retargeted, and new uses have been found using algorithms that integrate genomic and phenotypic data (Cheng et al., 2019; Kenneth and Cho, 2022). Most existing drug repositioning methods are limited to drug similarity networks and disease similarity networks, which do not account for information about cancer-related genes themselves or topological information between different biological networks (Cheng et al., 2019; Kenneth and Cho, 2022).

With the recent development of artificial intelligence and big data disciplines, an increasing number of machine learning (ML) and deep learning (DL) networks are being developed and used to analyze highly nonlinear network structures, such as gene, protein, disease, and drug networks (Angermueller et al., 2016; Ching et al., 2018). Using a network analysis model based on ML and DL, the combined analysis of genomics and systems biology networks makes it possible to identify druggable targets and perform drug repositioning while predicting cancer-related genes.

In this study, a target gene prediction–drug repositioning pipeline was constructed for LUAD. To more accurately predict potential drugs for the treatment of LUAD, target genes associated with LUAD were identified before drug prediction. Due to the complex network relationship between genes and the wide range of characteristics of the genes themselves, a graph neural network (GNN)-based graph attention network (GAT) model was built to be a gene classifier, which can predict genes that currently have unconfirmed relationship with LUAD are in fact associated with LUAD or not. Other models including a GNN-based graph convolutional network (GCN) model, a DL-based TabNet model, an ML-based random forest (RF) model (Ho, 1995), an Adaptive Boosting (AdaBoost) model (Freund et al., 1999), and an XGBoost classifier model (Chen and Guestrin, 2016) were built to compare their performances with the GAT model. The drug prediction performed in this study was based on the assumption that the target genes of a drug for the treatment of LUAD are related to the genes associated with LUAD. Under this assumption, two drug prediction methods, network overlap-based, and network distance-based methods were used to predict potential drugs for the treatment of LUAD.

## Materials and methods

### Data collection and pre-processing

Gene expression data were collected for 594 samples from The Cancer Genome Atlas (TCGA, https://www.cancer.gov/tcga) (Cancer Genome Atlas Research Network et al., 2013), of which 59 were precancerous tissue (normal) samples, and 535 were tumor samples. Genes were extracted from TCGA. To define LUAD-related target genes, the fragments per kilobase of exon per million mapped fragments (FPKM) values from the transcriptome profiling data were downloaded from TCGA. To further group genes and to identify genes that have statistically significant differences in expression in cancer tissue compared to normal tissue, differential expression analysis was performed. Specifically, fold change was calculated by dividing the mean FKPM in cancer tissue by the mean FPKM from normal tissue, thereby providing the fold change value for each gene. To facilitate differential expression analysis, log fold change was converted to log2fold change (log2FC), and adjusted *p*-value (false discovery rate, FDR) was calculated by limma package (v3.38.3) (Ritchie et al., 2015). Genes with an FDR ≥0.05 were excluded from the study. Genes with log2FC > 2 were classified as up-regulated genes, whereas those with log2FC < −2 were classified as down-regulated genes. Both up-regulated, and down-regulated genes were considered to be associated with LUAD. These LUAD-related genes were added to ‘‘positive’’ gene lists to facilitate subsequent model building. On the contrary, genes with log2FC > −0.3 and log2FC < 0.3 were considered not to be statistically differentially expressed in cancer tissue compared to normal tissue and were added to the “negative” genes list in the study. A Bayesian information criterion (BIC) method-based sensitivity analysis was performed to illustrate the rationale for the Log2FC threshold used in this study (Supplementary Material SB). The remaining genes were having an unconfirmed relationship with LAUD, and thus were added to the “ambiguous” genes list in the study. Positive and negative gene lists were used for GAT model training, validation, and testing, and the ambiguous gene list was used for potential LUAD-related target prediction. The model was designed to predict the association of genes with ambiguous relationships to LUAD (classified as LUAD-related or not).

To verify the predictive ability and validity of the model, a subset of genes was obtained from the Comparative Toxicogenomics Database (CTD) (Davis et al., 2020). The criteria of LUAD-related genes were: 1) Reported to have direct evidence of “marker/mechanism; ” 2) Had an Inference Score greater than 11.09 (75% quantile) or a Reference Count greater than 6 (75% quantile). The same number of genes that were reported to other diseases from CTD were randomly picked with the following criteria: 1) Not included in the training set or validation set of GAT; 2) Not included in LAUD-related gene list from CTD.

Gene function annotation analysis was performed using Gene Analytics (https://ga.genecards.org/) (Ben-Ari Fuchs et al., 2016) and VarElect (https://varelect.genecards.org/). *p*-values, matching scores, and average disease-causing likelihoods of gene-related tissues, systems, diseases, and pathways; gene ontology information; and human phenotype ontology information were collected, with a threshold *p*-value of 0.05. For convenient data processing, the original *p*-values were replaced by −log10 (*p*-values). Missing values were imputed with zeros to indicate that there was no association.

Gene function annotation features with greater than 40% of values missing, those with absolute Pearson’s correlation coefficient values greater than 0.98, and those that did not contribute to cumulative importance of 0.99 were excluded from the analysis. Samples were divided into training (80%), validation (10%), and testing sets (10%) using a stratified sampling method. The imputation of missing values and data normalization were performed on the training, validation, and testing sets separately after dataset division to avoid data leakage.

Gene-gene interaction information was collected from the Human Reference Interactome (http://www.interactome-atlas.org/) (Luck et al., 2020) ,RNAInter v4.0 (http://www.rnainter.org/) (Kang et al., 2022), and the human protein-coding Gene Functional Association Network. The high-confidence gene associations from different databases were merged, and gene-gene interaction subgraphs were constructed according to the genes required for the study. Functionally related protein-coding genes, RNA-RNA associations, and RNA-protein associations were recorded as the background gene-gene interaction network of this study. As the interaction edges from the Human Reference Interactome were un-weighted, the unweighted interactions were replaced by the average edge weight of the subgraph. The weights of all edges are input into the network as initial values. During the learning process of the network, the weights of each edge are iteratively updated. Drug target genes were collected from PharmGKB (https://www.pharmgkb.org/) (Whirl-Carrillo et al., 2021), the Binding Database (https://www.bindingdb.org/) (Liu et al., 2007), and DrugBank (https://go.drugbank.com/) (Wishart et al., 2018). Data from human subjects collected and used in this research project have been approved by the University’s Research Ethics Subcommittee. Data is available at: https://github.com/Clement-HUANG/LUAD-Drug-Repositioning. The hyperparameters and sought ranges of trained models in this study are available in the Supplementary Table S1.

### Target gene prediction model development and evaluation

A GAT model was developed for LUAD-related target gene prediction. During the construction of the graph, genes were input as nodes, and the features of the nodes were high-dimensional gene function annotation features. The edges of the graph were the gene-gene associations, and the weights of the edges were the weights of the association. The three-fold cross-validation method was used to obtain optimal model parameters, and “auc” was used as the model metric. The random search method was applied for the approximate range of hyperparameters, and the grid search method was applied afterward to search for the best hyperparameters in a small range. The trained GAT model was used to analyze the “ambiguous” gene list for the final LUAD-related target gene prediction. ML-based models, including RF, AdaBoost, and XGBoost classifier models; a DL-based TabNet model; and a GNN-based GCN model were built to compare their performance with the GAT model. A support vector machine classifier and a logistic regression model were also built but were excluded from the comparison as they were time-consuming to execute, and they showed low precision for processing high-dimensional data. Gene enrichment analysis was applied for LUAD-related genes identified by differential gene expression analysis and LUAD-related genes predicted by the GAT model. To extend the GAT model established in this study, additional experiments were applied to test the ability of the GAT model to predict genes associated with enriched GO terms and KEGG pathways. In addition, the gene interaction networks of BioGRID (Stark et al., 2006), BioSNAP (PP-Pathways), and BioSNAP (TFG-Ohmnet) (Leskovec and Sosič, 2016) were collected to test the sensitivity of the GAT model to different gene interaction networks. The GAT-trained gene-gene interaction network was derived, and the genes were clustered using the K-mean clustering method. This method’s optimal K value (the number of clusters) was determined by the silhouette index (Rousseeuw, 1987), which described the node’s association and its corresponding cluster. To better interpret this network, gene enrichment analysis using the DAVID online database (Huang Da et al., 2009; Sherman et al., 2022) was applied to the genes of each cluster.

### Drug repositioning module

The drug repositioning module was based on the LUAD-related gene interaction network and drug-gene interaction network. Among them, the LUAD-related gene network was built according to LUAD-related genes and their associated genes from the original gene-gene interaction network; and the drug-gene network was constructed according to drug target genes and their directly related genes from the original gene-gene interaction network. The module was mainly composed of the network overlap-based drug prediction model, the network distance-based drug prediction model, and the summary model. The Jaccard score is a statistic that measures the similarity between two sample sets. In the network overlap-based drug prediction model, the similarity between the LUAD-related target gene networks and the drug target gene networks was calculated using the Jaccard score as the measure (Bass et al., 2013). The Jaccard score between the LUAD-related target gene networks and lists (N_g_) and the i^th^ drug target gene network (N_di_) was calculated according to Eq. 1.

$$J\left(N_{g},N_{\mathrm{di}}\right)=\;\frac{\left|N_{g}\cap N_{\mathrm{di}}\right|}{\left|N_{g}\cup N_{\mathrm{di}}\right|}=\;\frac{\left|N_{g}\cap N_{\mathrm{di}}\right|}{\left|N_{g}\right|+\left|N_{\mathrm{di}}\right|-\left|N_{g}\cap N_{\mathrm{di}}\right|}$$ (1)

The network distance-based drug prediction model calculated the length of the shortest path (d) from the genes (g) in the LUAD-related target gene network (N_g_) to the i^th^ drug (N_di_) in the target gene network (N_d_) according to Eq. 2.

$$d\left(N_{g},N_{\mathrm{di}}\right)=\frac{1}{\left|\left|N_{d}\right|\right|}\sum_{N_{\mathrm{di}}}\mathrm{mindistance}\left(g,N_{\mathrm{di}}\right)$$ (2)

The z-score was used to standardize the distance between N_g_ and N_di_. It was calculated based on the shortest path length (d) and it’s mean ( $\mu_{i}$ ) and the standard error ( $\sigma_{i}$ ) of the null distribution according to Eq. 3.

$$z-\mathrm{score}=\;\frac{d\left(N_{g},N_{\mathrm{di}}\right)-\mu_{i}\;}{\sigma_{i}}$$ (3)

Jaccard scores and z-scores for the drugs were calculated using the online tool PharmOmics (http://mergeomics.research.idre.ucla.edu/) (Chen et al., 2022) and were included in the final summary model. Drugs with a Jaccard score more than the mean value of all the drugs, and a z-score less than −2 were output as potential therapeutics for LUAD (one side *p*-value < 0.05). Based on the difference between the values for N_g_ and N_di_, drugs with an overlap (overlapped genes/N_di_) < 50% were excluded.

### *In-silico* validation of predicted drugs

To validate the affinities of potential drugs, and preclinical compounds predicted from this study with their target proteins, the half-maximal inhibitory concentration (IC50) values were collected from Binding Database. The drug-target protein pairs with median IC50 values less than 10,000 nM were considered as having physical drug-target interactions (Cheng et al., 2019).

## Results

**FIGURE 1:**
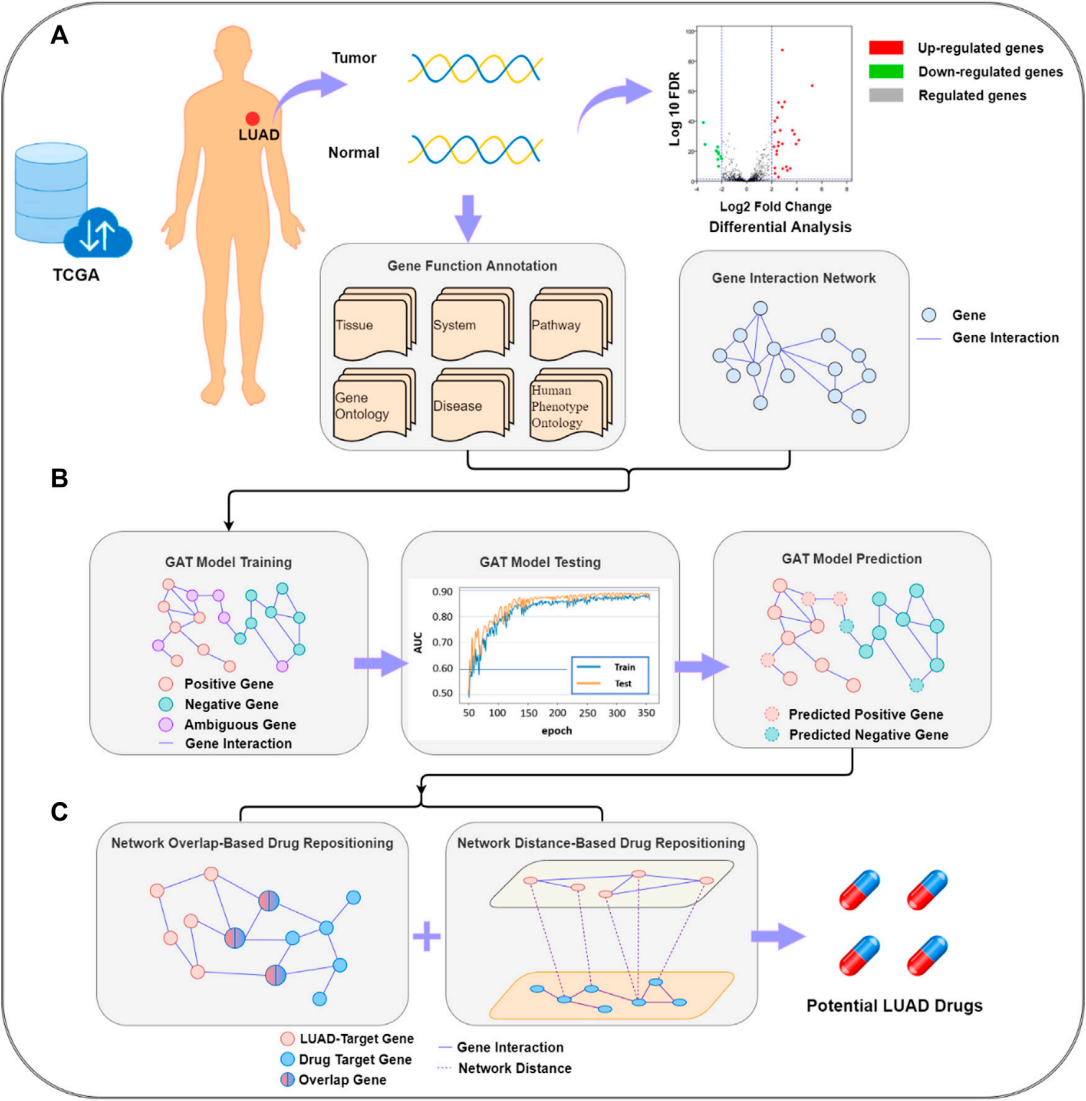
Lung adenocarcinoma target gene prediction–drug repositioning pipeline. **(A)** data collection and pre-processing; **(B)** graph attention network (GAT) model building and lung adenocarcinoma (LUAD)-related target gene prediction; **(C)** potential LUAD drug prediction. FDR, false discovery rate; TCGA, The Cancer Gene Atlas.

The target gene prediction–drug repositioning pipeline is shown in Figure 1. LUAD target gene prediction was based on genes, gene interaction networks, and functional annotation data. Through the established GAT, potential LUAD-related target genes were predicted. The target genes were passed into the drug repositioning module as the input of the drug redirection network. The module was a double-layer structure. The first layer was composed of a network overlap-based drug prediction model and a network distance-based drug prediction model, and the second layer was a summary of the results of the two models.

### Data pre-processing

**FIGURE 2:**
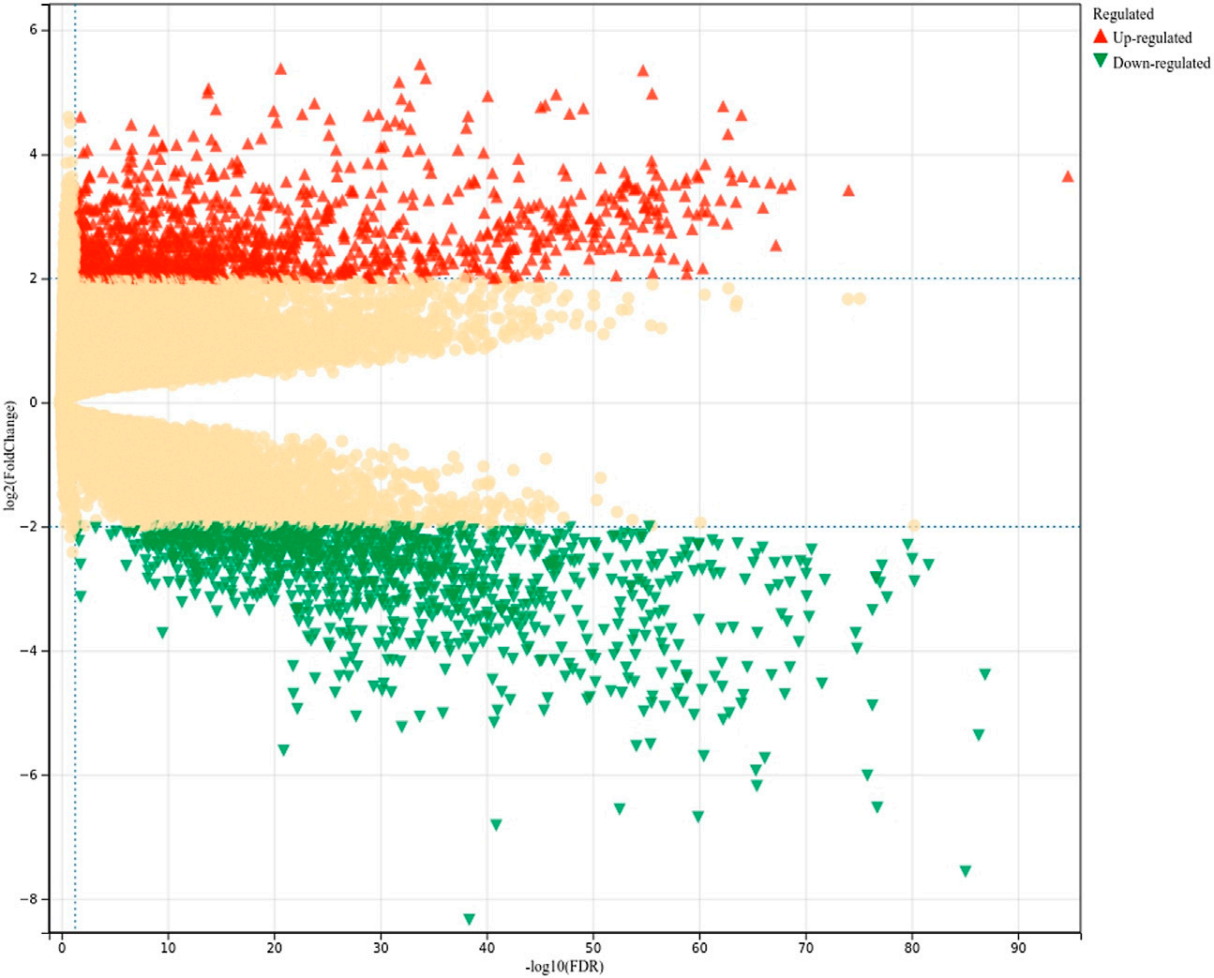
Analysis of differentially expressed LUAD-related genes. FDR, false discovery rate.

Genes were extracted from TCGA. The FPKM data for the genes were used for differential gene expression analysis (Figure 2). Of the 48,597 genes (19,054 protein-coding genes; 29,543 non-coding genes) included in the analysis, 19,730 had an FDR <0.05, 1,119 were upregulated, and 1,064 were downregulated. After excluding pseudogenes, the remaining 1,946 dysregulated genes were classified as positive genes (LUAD-related target genes) for model training. The absolute value of log2 fold change (log2FC) was <0.3 for 1,379 genes, and these genes were considered to be irrelevant to LUAD and thus were used as negative genes for training. The remaining 13,062 genes were classified as having an unclear relationship with LUAD, awaiting prediction by the model.

One hundred and fifty functional annotation features (tissues, systems, diseases, and pathways; gene ontology information; and human phenotype ontology information) of the genes were collected, and suitable features were selected to train the model. Features with greater than 40% of values missing and those with absolute Pearson’s correlation coefficient values greater than 0.98 were removed from the analysis due to low quality and high redundancy, respectively. Sixty-eight features were selected and used for subsequent model training. The 15 most significant prediction features with their normalized importance scores and the number of features with a cumulative importance score greater than 0.99 are shown in Supplementary Figure S1. Of all the gene functional annotation features, the importance score of gene enrichment significance was highest in the nervous system (normalized importance coefficient >0.05). The enrichment significance of genes in lung, brain, blood, kidney, and epithelial cells also had a high ranking (>0.03). In terms of disease enrichment features, genes were more significantly enriched in breast cancer than in lung cancer. The enrichment significance of pathways and gene ontologies related to lung cancer did not rank within the top 15.

### Performance of the GAT model

**FIGURE 3:**
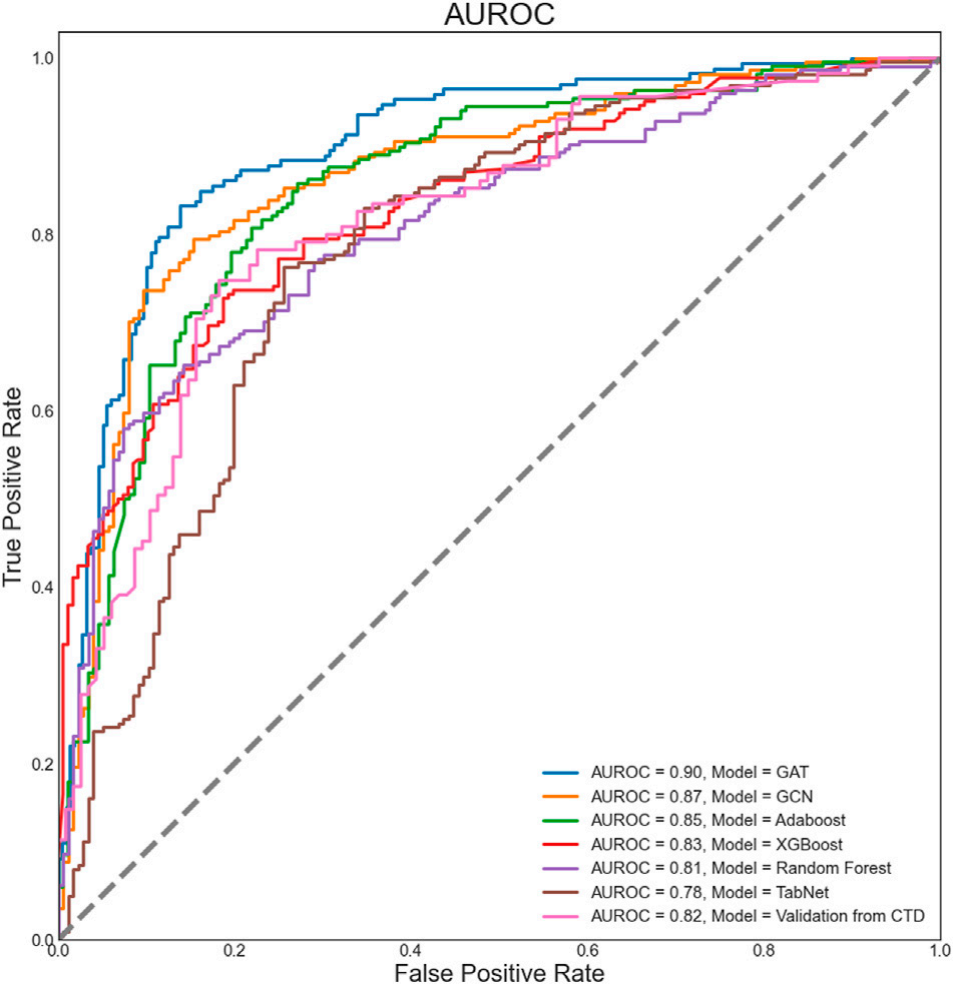
Areas under the receiver operating characteristic curves of the models. AUROC, area under the receiver operating characteristic curve; GAT, graph attention networks; GCN, graph convolutional network.

The models’ power to predict LUAD-related target genes was determined based on the area under the receiver operating characteristic curve (AUROC), as shown in Figure 3. The GAT model achieved the best prediction performance in the internal data tests, reaching the highest AUROC value (0.90). The GNN-based GCN, ML-based RF, XGBoost, and Adaboost models also achieved good prediction performance (AUROC = 0.87, 0.81, 0.83, and 0.85, respectively). However, the DL-based TabNet model did not show good performance, with an AUROC value of less than 0.8. The external validation dataset from CTD achieved an AUROC of 0.82. In addition to AUROC, model evaluation metrics such as precision, recall, and F1-score were also used to measure the predictive ability of the model (Table 1). GNN-based GAT and GCN models achieved above 0.80 in precision, recall, and F1-score, which were better than DL-based TabNet, ML-based XGBoost, RF, and Adaboost. GAT achieved the highest precision, recall, and F1-score (precision = 0.85, recall = 0.85, F1-score = 0.85), indicating its strong classification ability for positive and negative samples. The precision, recall, and F1-score of the external validation dataset from CTD all achieved 0.78. 134 GO terms and KEGG pathways were enriched by LUAD-related genes (FDR<0.05), including 69 GO biological process terms, 27 GO cellular component terms, 28 GO molecular function terms, and 10 KEGG pathways. The top 5 terms of each category, together with the prediction AUROC and AUPRC, were reported according to the order of FDR (Supplementary Table S2).

**TABLE 1:** Model evaluation metrics: AUROC, precision, recall, and F1 score.

Model name	AUROC	Precision	Recall	F1-score
GAT	0.90	0.85	0.85	0.85
GCN	0.87	0.82	0.82	0.82
Adaboost	0.85	0.79	0.79	0.79
XGBoost	0.83	0.77	0.76	0.76
Random forest	0.81	0.77	0.74	0.74
TabNet	0.78	0.75	0.75	0.75
CTD validation	0.82	0.78	0.78	0.78

The prediction of LUAD-related genes in this study was sensitive to the gene interaction network. The results and prediction performance of loading different gene interaction networks into the GAT model were not completely consistent, which might be caused by various gene interaction network sources. In addition to the results reported in Table 1, the GAT model achieved an AUROC of 0.85 using the gene interaction network from the Biogrid database; an AUROC of 0.87 using the gene interaction network from the BioSNAP database; and in particular, an AUROC of 0.85 using the lung tissue-related gene interaction network from the BioSNAP database (Supplementary Table S3). Although good LUAD-related gene prediction ability can be achieved using lung tissue-related gene interaction networks, it might be a risk to be applied in drug repositioning analysis, because drug genes are not necessarily associated with lungs only and vice versa.

### Newly predicted LUAD-related target genes

After training the GAT model, genes with ambiguous relationships with LUAD were input into the trained GAT model to identify more LUAD-related genes. The output of the GAT model was the likelihood (probability) that a gene was a LUAD-related target gene, with values ranging from 0 to 1. Genes with an output probability value closer to 1 had a stronger association with LUAD. The Youden index of AUROC was calculated to find the optimal threshold for the model. The genes with an output higher than the optimal threshold can be considered to be associated with LUAD. The optimal threshold of the GAT model was 0.664, and genes with scores >0.664 were all potential LUAD-related genes originally. To simplify the complexity of the drug prediction network and improve the computational speed, the genes with output scores higher than 0.9 were used as the predicted LUAD-related genes for subsequent drug prediction. Using a threshold between 0.664 and 0.9 increased the complexity of the drug prediction network and the computation time of the Jaccard score and z-score, but not able to identify more drugs that reached a statistically significant level. Of the 10,858 genes identified as having an ambiguous relationship with LUAD, 1,597 genes had an output score >0.9 and were considered to be potential target genes for LUAD treatment.

**FIGURE 4:**
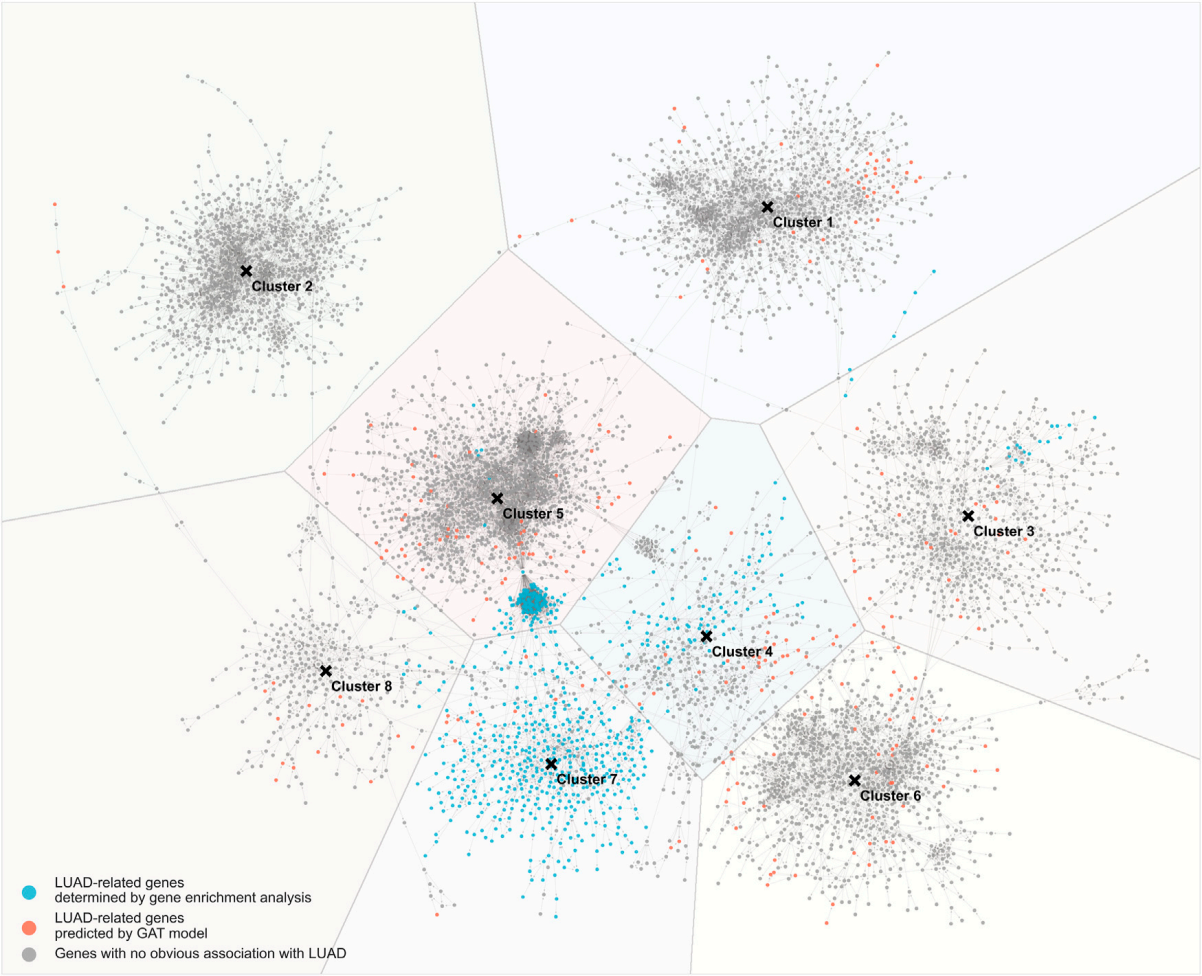
Gene-gene interaction network trained by GAT model. Blue nodes: LUAD-related genes determined by gene enrichment analysis; red nodes: LUAD-related genes predicted by GAT model; grey nodes: genes with no obvious association with LUAD.

Based on the results of GAT model training, the interaction network between genes was drawn (Figure 4). Among them, the blue nodes are LUAD-related genes identified by the gene differential expression analysis, the red nodes are LUAD-related genes predicted by the GAT model, and the grey nodes are LUAD-unrelated genes. The distance between nodes is determined by the strength of the interaction between genes, and two nodes with close distances indicate strong interactions between the two genes. In the gene-gene interaction network, 8 clusters of genes were found. The number of clusters is determined from the optimal silhouette index of the K-mean clustering method. Among the 8 clusters, the 7th cluster contained more LUAD-related genes (>50%). To further investigate the genes network, gene enrichment analysis using the DAVID online tool was applied to the genes of each cluster. The enrichment of genes in each cluster was assessed according to the top 5 GO terms from low to high FDR (threshold FDR <0.05), and the enriched KEGG pathways are reported (Supplementary Table S4).

### Prediction of potential therapeutic drugs for LUAD

The original positive samples and the potential target genes for LUAD treatment predicted by the GAT model were integrated and input into the drug prediction module, along with 3,070 drugs approved by the Food and Drug Administration and 672 preclinical compounds for use in humans. In the overlap-based drug prediction network, Jaccard scores were calculated to represent the degree of overlap between drug target genes and LUAD-related genes. The Jaccard scores for all drugs were generally normally distributed (Supplementary Figure S2A), with a mean of 0.036 and a standard deviation of 0.011. In the network distance-based drug prediction network, the z-score of each drug was calculated to represent the distance between the drug target gene network and the LUAD target gene network. A smaller z-score represented a closer distance. The z-score distribution of the individual drugs was not normal (Supplementary Figure S2B), with a mean of -3.848 and a standard deviation of 2.951. In total, 1,855 drugs and preclinical compounds from the overlap-based network had Jaccard scores higher than the mean Jaccard score, and 35 drugs or preclinical compounds had z-scores less than -2 and were thus considered to be potentially associated with LUAD.

The prediction results of the two networks were summarized, and drugs with a drug-gene overlap rate of less than 50% were eliminated. Ten drugs or compounds met all the requirements and were predicted to be potential drugs for preclinical studies of LUAD (Table 2). The predicted results were compared with data from the DrugBank database, the CTD, and literature searches of PubMed. Of the ten predicted drugs and preclinical compounds, three were new findings and had not previously been reported. Of the remaining seven, five were in the laboratory stage (*in vitro*), one was entering the clinical trial stage (phase 2), and one was reported to be discovered (reported) with experiments under preparation.

**TABLE 2:** Potential drugs or preclinical compounds for lung adenocarcinoma.

Drug/preclinical compound	Jaccard score	Z-score	Validation	Reference
Chlorpromazine	0.059	−2.371	*In Vitro*	(Zhu et al., 1991; Ogretmen et al., 2002)
Bromoethylamine	0.057	−2.476	NA	-
Azathioprine	0.054	−3.848	Reported	(McAdam et al., 1974; Lazarev et al., 2012; Baik et al., 2018)
Ethanol	0.050	−2.310	*In Vitro*	Chen et al., 2012
Papaverine	0.044	−3.345	*In Vitro*	Gomes et al., 2021
Fluoxetine	0.043	−3.516	Phase 2	Yang et al., 2021
Cimetidine	0.040	−2.125	NA	-
Benzbromarone	0.039	−2.325	NA	-
Rotenone	0.038	−3.437	*In Vitro*	Shi et al., 2014
Sulfasalazine	0.038	−2.663	*In Vitro*	Hu et al., 2020

There were 47 binding target proteins from the three potential drugs predicted from this study, and 14 of them were identified from GAT and with an IC_50_ value less than 10,000 nM, which presents the physical interaction between drugs and targets (Table 3). The proteins and genes that were not found by GAT or IC_50_ larger than 10,000 nM were listed in (Supplementary Table S5).

**TABLE 3:** Physical drug-target interaction pairs and IC_50_ values.

Drugs	Target name	Gene symbol	IC_50_ (nM)	Cell line
Benzbromarone	Cytochrome P450 2C9	CYP2C9	41.00	*Cebus apella*, and HRPTEpiC cells
	Aldo-keto reductase family 1 member C1	AKR1C1	48.00	BAOEC cells
	Solute carrier family 22 member 6	SLC22A6	4,600.00	*Drosophila* S2 cells
Cimetidine	Histamine H2 receptor	HRH2	500.00	U2OS cells
Bromoethylamine	Neuropeptide Y receptor type 1	NPY1R	0.06	SK-N-MC cells
	G protein-coupled receptor kinase 5	GRK5	11.80	In-silico study (3D-QSAR)
	Matrix metalloproteinase-9	MMP9	26.00	Kinetic study
	Anoctamin-1	ANO1	156.50	FRT, and U251 cells
	Protein farnesyltransferase chain B	FNTB	180.00	NIH3T3 cells
	Delta-type opioid receptor	OPRD1	362.00	CHO cells
	DNA repair protein RAD51 homolog 1	RAD51	370.00	HEK293 cells
	ATP-binding cassette sub-family G member 2	ABCG2	527.50	H460/MX20 cells
	Thymidylate synthase	TYMS	1,288.00	In-silico study (3D-QSAR)
	Dihydrofolate reductase	DHFR	5,010.00	HL-60, Bel-7402, BGC823, KB, Hela, and SK-OV-3 cells

## Discussion

In this study, a LUAD-related target genes and drug prediction pipeline was developed. A GAT model was used for target gene prediction, and an overlap-based network and a network distance-based methods were used for LUAD drug prediction. Using this pipeline, together with FPKM data from TCGA, a gene interaction network, gene function annotation features, and drug target gene information, 1,597 potential LUAD-related target genes and 10 potential drugs or preclinical compounds for the treatment of LUAD were predicted. The AUROC of the GAT model developed in this study reached 0.89, which indicates the robust prediction of LUAD-related target genes.

Due to the absence of standard datasets of LUAD-related and -unrelated genes (Schulte-Sasse et al., 2021), we used a more stringent log2FC criterion than conventional studies. An absolute value of log2FC greater than 2 was required for a gene to be considered to be related to LUAD, while traditionally, an absolute value of log2FC greater than 1 is used as the threshold. A stricter threshold selection is beneficial to reduce the error of the sample data in the training set and thus achieve more accurate predictions. As few datasets reported genes unrelated to cancer, genes with an absolute value of log2FC close to 0 were classified as unrelated genes.

We also compared the prediction performance of the GAT model with the prediction performance of traditional ML and DL models. Compared with the TabNET model, the ML-based decision tree model had better prediction performance. There are two possible reasons for this. First, the training sample size and the number of features were small (Kotsiantis, 2007). There were only approximately 3,000 genes and less than 100 features in the training set. Within the scope of this analysis, ML-based decision trees had obvious advantages. Second, the TabNet model is not a GNN-based model, and therefore, it is not conducive to summarizing topological information from gene interaction networks. Compared with ML-based decision trees, deep learning models (GAT and GCN) based on GNN had better prediction performance. GNNs are applied to graphs in the non-Euclidean space. Non-Euclidean spaces represent more arbitrary spaces than Euclidean spaces due to arbitrary connections between nodes, thus allowing GNNs to obtain topological information more effectively. For feature extraction, the analytical ability of the GNNs was close to that of the ML-based decision tree. Both GCN and GAT are types of GNNs, and both methods aggregate the features of neighboring vertices to the central vertex and use the local stationary points on the graph to learn new vertex feature expressions. The difference is that GCN uses the Laplacian matrix, whereas GAT uses the attention coefficient. To a certain extent, GAT models are more precise as the correlation between vertex features is better incorporated into the model (Li et al., 2015). Due to the difference in the vertex operation mode, changing the structure of the graph in the test task has little impact on the GAT. In the training process of most graph neural networks, the training set is only a subgraph of the whole graph, and the testing set is a new subgraph for the model. GCN is a calculation method for the entire graph, and the node features of the entire graph are updated in one calculation, which makes the GCN model inefficient and poor in handling unknown subgraphs (Kipf and Welling, 2016). The learned parameters in GCNs are primarily related to the graph structure. On the other hand, for the GAT model, the new subgraph only affects the relationship matrix of nodes to feature mapping. The dimension of this matrix is determined at the beginning of the model establishment and is only related to the number of features of each node. Therefore, GATs are more effective than GCN at inductive tasks, such as supervised learning (Hamilton et al., 2017).

In the drug prediction module, overlap-based and network distance-based networks are commonly used prediction methods, and several drug prediction models have been built using similar theories (Tu, 1996; Huang et al., 2016; Li et al., 2020; Jain et al., 2021). As there are no data on which type of predictive model is more reliable, both predictive models were used in this study and then summarized to predict potential drugs for the treatment of LUAD.

Among the ten predicted drugs or preclinical compounds, three were newly discovered using this prediction pipeline. The remaining eight drugs have been previously identified as potential drugs for the treatment of LUAD, and some are already in the laboratory testing stage or clinical trial stage. Bromoethylamine is a new potential preclinical compound for LUAD treatment identified in this study (Jaccard score = 0.057, z-score = -2.476). An association between bromoethylamine and LUAD has not been reported in DrugBank, the CTD, or in previous studies. Notably, in the CTD, bromoethylamine is reported to interact with hepatitis A virus cellular receptor 1 and natriuretic peptide receptor 1, which coincide with the genes predicted in this study. Additionally, both genes are associated with fibrosis (Cherngwelling et al., 2021; Priego et al., 2021), which is an important phenotype that occurs throughout the progression of LUAD (Angel et al., 2020). Moreover, these two genes also show strong associations with kidney-related diseases (Kanki et al., 2014).

Bromoethylamine is used to synthesize anti-radon, a brain drug. Anti-radon has a central excitatory effect. It promotes the release of active sulfhydryl groups in the body and participates in the redox process of brain cells, thereby promoting and restoring the metabolism of brain cells to enable the rapid restoration of brain function in traumatic coma patients. In addition, there are anti-central nervous system depressant drugs that are clinically suitable for traumatic coma, coma caused by other reasons, the sequelae of traumatic brain injury, carbon monoxide poisoning, cerebral hypoxia, and sleeping pill poisoning (Sun and Li, 2008). Cimetidine (Jaccard score = 0.040, z-score = −2.125) was also predicted to be a potential treatment for LUAD. Although the use of cimetidine for the treatment of NSCLC has been assessed in clinical trials, it did not appear directly as a LUAD-related drug. However, it was used as an allergy prevention drug in a clinical trial of paclitaxel for the treatment of NSCLC (Hainsworth and Greco, 1994). Currently, cimetidine is used to treat gastroesophageal reflux disease, peptic ulcer disease, and dyspepsia. It is also being tested in clinical trials for the treatment of chronic obstructive pulmonary disease and community-acquired pneumonia. The target genes of cimetidine include Epidermal Growth Factor Receptor (*EGFR*) and Aryl Hydrocarbon Receptor (*AHR*), both of which are LUAD-related genes. Benzbromarone is another drug newly identified in this study as having potential for the treatment of LUAD (Jaccard score = 0.039, z-score = −2.235). Benzbromarone has been studied for its effects on the development of hepatoma and metabolic pathways of lipids, proteins, amino acids, and their derivatives (Calvisi et al., 2011). It has also been used in trials studying the basic science and treatment of heart failure, hyperuricemia, chronic kidney disease, abnormal renal function, gout, and asymptomatic hyperuricemia. Benzbromarone is currently being assessed in the clinical trials for the treatment of heart failure/hyperuricemia, type 2 diabetes, and pulmonary idiopathic arterial hypertension. No previous studies have linked benzbromarone with LUAD.

The seven remaining drugs have previously been reported to be LUAD-related or have entered clinical trials for LUAD treatment. For example, chlorpromazine, which was predicted in this study (Jaccard score = 0.059, z-score = −2.371), is a phenothiazine anti-psychotic used to treat nausea, vomiting, preoperative anxiety, schizophrenia, bipolar disorder, and severe behavioral problems in children. Chlorpromazine has been reported to modulate the metabolism of papillary lung adenocarcinoma cells by targeting c-Myc (Ciribilli et al., 2016). In addition, in cell line experiments, chlorpromazine has been shown to be effective at inhibiting the growth of lung tumor cell lines (the human small cell carcinoma-derived cell line IRSC-10M and the adenocarcinoma-derived cell line A549) (Zhu et al., 1991). Fluoxetine (Jaccard score = 0.043, z-score = −3.516) was originally developed as an anti-depressant in the selective serotonin reuptake inhibitor class (March et al., 2004). It is commonly used to treat depression and sometimes obsessive-compulsive disorder and bulimia (Husted et al., 2007). In addition, fluoxetine is also a promising drug for treating patients with depression and NSCLC (Yang et al., 2021). The use of this drug for the treatment of a variety of disorders may be related to the significant expression levels of LUAD-related genes in the nervous system (Hsu et al., 2020), which was also partially confirmed by the feature selection in this study. This finding of nervous system-related drugs, including selective serotonin reuptake inhibitors, being potentially suitable for treating lung cancer is not an independent event, as aducanumab, a drug for Alzheimer’s disease (Schneider, 2020), has also been experimentally tested for lung cancer and encouraging results were observed (Morrison, 2016).

Azathioprine has previously been reported to be used for treating patients with LUAD, but its effect and mechanism of action are unknown (McAdam et al., 1974; Lazarev et al., 2012; Baik et al., 2018). Ethanol has the potential to suppress CL1-5 human LUAD cell migration, and it inhibits matrix metalloproteinase-2/9 via the extracellular signal-regulated kinase, c-Jun N-terminal kinase, p38, and phosphatidylinositol 3-kinase/Akt signalling pathways (Chen et al., 2012). A clinical trial of ethanol for the treatment of LUAD is currently in the recruiting stage. Papaverine may be used to treat various types of smooth muscle spasms, including vasospasm and visceral spasm associated with acute myocardial infarction and angina pectoris. It is currently being assessed in a clinical trial for the treatment of NSCLC. Rotenone has the potential to sensitize NSCLC cell lines to tumor necrosis factor-related apoptosis-inducing ligand-induced apoptosis (Shi et al., 2014), but it has not been assessed in a clinical trial, possibly because of its potent toxicity. Sulfasalazine is an anti-inflammatory drug used to treat Crohn’s disease and rheumatoid arthritis. Previous studies have shown that sulfasalazine abolishes the phosphorylation of AXL and other receptor tyrosine kinases, thereby reducing LUAD metastasis and drug resistance (Lay et al., 2007). No clinical trials using sulfasalazine for the treatment of LUAD have been conducted.

The present study has some limitations. The drug prediction pipeline is not able to distinguish between drugs that are used to relieve the symptoms of LUAD and those that eradicate LUAD. The reason is that the GAT model established in this study only screens out the genes related to LUAD, and it cannot determine whether the abnormal expression of genes occurs before or after the onset of LUAD. Therefore, it cannot be determined whether the predicted drug targets a gene involved in the development of LUAD, a gene that is abnormally expressed after the development of LUAD, or both. However, if LUAD-related oncogenes can be identified using statistical-based Mendelian randomization analysis or cellular or biological assays, potential drugs for the treatment of LUAD may be more targeted. In addition, the node attention coefficients in the GAT model of this study and the weights of edges between nodes may have potential implications for the prediction of new gene associations.

## Conclusion

In this study, a GAT-based target gene prediction–drug repositioning pipeline was constructed for LUAD. Using this pipeline, 1,597 genes were predicted as potential target genes for LUAD, bromoethylamine was predicted as a novel potential compound for the treatment of LUAD, cimetidine and benzbromarone were predicted as potential therapeutic drugs for LUAD. In the future, it is a potential research direction to use the attention coefficients and edge weights obtained in the GAT model to predict new gene-gene interaction relationships. Introducing cell line experiments and animal experiments to verify the efficacy of treating LUAD with bromoethylamine, cimetidine, and benzbromarone will also be a key research direction.

## Data Availability

The original contributions presented in the study are included in the article/Supplementary Material, further inquiries can be directed to the corresponding author.
