# Supplementary material for: Lung adenocarcinoma-related target gene prediction and drug repositioning
Source: Front Pharmacol. 2022 Aug 23;13:936758. doi: 10.3389/fphar.2022.936758 (PMC9445420; doi:10.3389/fphar.2022.936758)
Supplement: Supplementary file 1 [file DataSheet1.docx]

Supplementary Material

Part A:

# Supplementary Figures and Tables


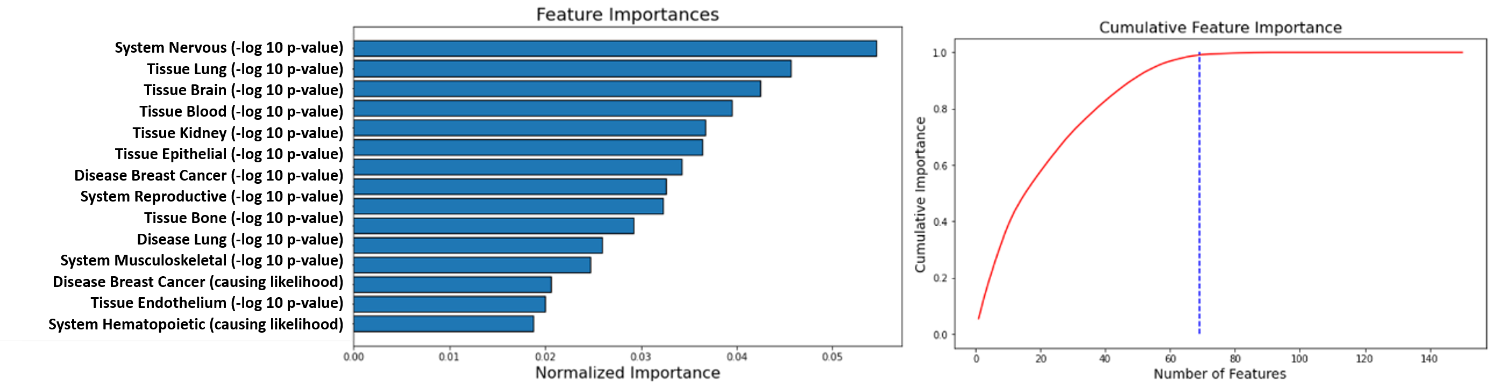


**Supplementary Figure 1.** Feature Selection of GAT Model. A total of 68 features were selected and used for subsequent model training. The top 15 key prediction features with their normalized importance scores (left) and the number of features with cumulative importance larger than 0.99 (right) are shown.


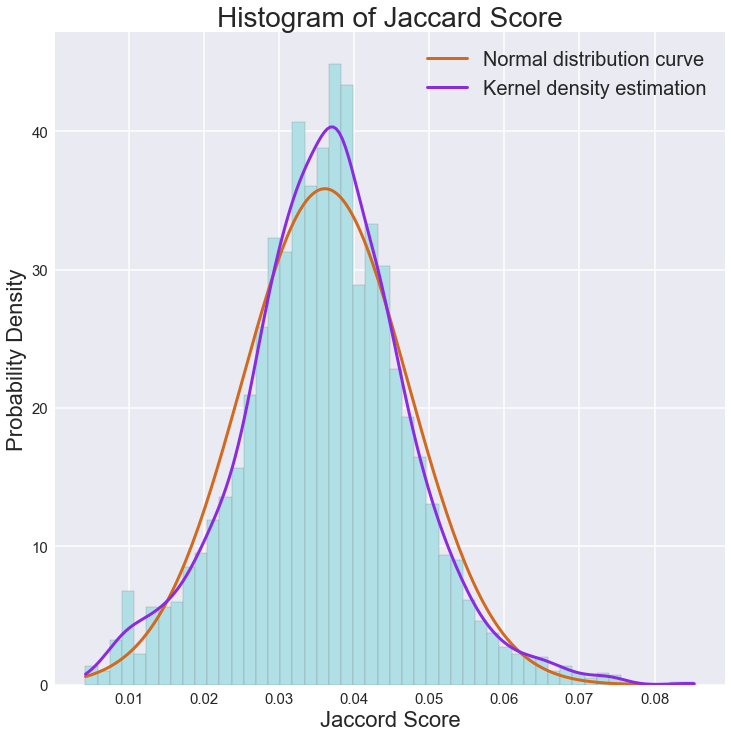


**Supplementary Figure 2a.** Distribution of Jaccard Score and Z-score. (a) Histogram of Jaccard Score.


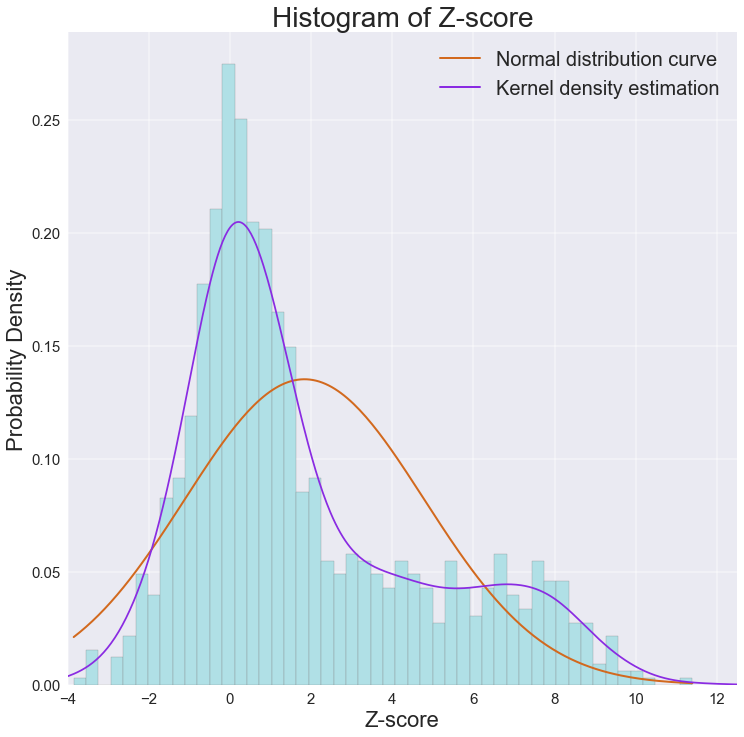


**Supplementary Figure 2b.** Distribution of Jaccard Score and Z-score. (b) Histogram of Z-score.

**Supplementary Table 1.** Trained hyperparameters in this study.

| **Model** | **Hyperparameter Name** | **Sought Range** |
| --- | --- | --- |
| **GAT** | Layer sizes | 5-15 |
|  | Activations | Tanh, relu, softmax |
|  | Attention heads | 2-20 |
|  | Inside dropout rate | 0.05-0.9 |
|  | Attention dropout rate | 0.05-0.9 |
|  | Optimizer | Adam (learning rate:0.01-0.05) |
|  | Loss | Binary cross-entropy |
| **GCN** | Layer sizes | 5-15 |
|  | Activations | Tanh, relu, softmax |
|  | Optimizer | Adam (learning rate:0.01-0.05) |
|  | Loss | Binary cross-entropy |
| **AdaBoost** | Max depth | 3-15 |
|  | Min samples leaf | 3-10 |
|  | Min sample split | 2-50 |
|  | Learning rate | 0.01-0.8 |
|  | Number of estimators | 100-500 |
| **XGBoost** | Learning rate | 0.01-0.05 |
|  | Objective | binary: logistic |
|  | Number of estimators | 50-500 |
|  | Max depth | 3-15 |
|  | Min child weight | 1-5 |
|  | Gamma | 0-0.2 |
| **Random Forest** | Max depth | 3-21 |
|  | Max features | 3-11 |
|  | Min samples leaf | 1-60 |
|  | Min samples split | 5-15 |
|  | Number of estimators | 10-150 |
|  | Oob score | True |
| **TabNet** | Depth | 16-64 |
|  | Gamma | 0.8-1.5 |
|  | Number of steps | 2-5 |
|  | Optimizer | Adam (learning rate:0.01-0.05) |
|  | Verbose | 1 |

**Supplementary Table 2.** Top 5 terms from GO terms and KEGG pathways of LUAD-related genes (FDR<0.05) with prediction AUROC and AUPRC.

| **Enrichment Analysis** | **Description** | **AUROC** | **AUPRC** |
| --- | --- | --- | --- |
| Biological Process (GO) | Cell adhesion | 0.856 | 0.836 |
|  | Extracellular matrix organization | 0.756 | 0.727 |
|  | Homophilic cell adhesion via plasma membrane adhesion molecules | 0.852 | 0.830 |
|  | Proteolysis | 0.791 | 0.754 |
|  | Negative regulation of endopeptidase activity | 0.830 | 0.801 |
| Cellular Component (GO) | Extracellular region | 0.954 | 0.946 |
|  | Extracellular space | 0.884 | 0.892 |
|  | Integral component of plasma membrane | 0.945 | 0.916 |
|  | Extracellular matrix | 0.849 | 0.802 |
|  | Plasma membrane | 0.830 | 0.823 |
| Molecular Function (GO) | Serine-type endopeptidase activity | 0.904 | 0.878 |
|  | Calcium ion binding | 0.790 | 0.758 |
|  | Extracellular matrix structural constituent | 0.929 | 0.927 |
|  | Metalloendopeptidase activity | 0.764 | 0.664 |
|  | Serine-type endopeptidase inhibitor activity | 0.941 | 0.938 |
| Pathway (KEGG) | Neuroactive ligand-receptor interaction | 0.999 | 0.999 |
|  | Complement and coagulation cascades | 0.984 | 0.984 |
|  | Protein digestion and absorption | 0.930 | 0.920 |
|  | ECM-receptor interaction | 0.969 | 0.944 |
|  | Cell adhesion molecules | 0.980 | 0.979 |

**Supplementary Table 3.** Model evaluation for different protein-protein interaction databases.

| **Model Name** | **AUROC** | **Precision** | **Recall** | **F1-Score** |
| --- | --- | --- | --- | --- |
| **GAT** | 0.90 | 0.85 | 0.85 | 0.85 |
| **BioGRID** | 0.85 | 0.79 | 0.80 | 0.79 |
| **BioSNAP** | 0.87 | 0.82 | 0.82 | 0.82 |
| **BioSNAP**  **(Lung specific)** | 0.85 | 0.81 | 0.80 | 0.81 |

**Supplementary Table 4.** Subgraph enrichment analysis displayed the top 5 GO terms and KEGG pathways with FDR < 0.05 in an ascending order.

|  | **Enrichment Analysis** | **Description** |
| --- | --- | --- |
| **Cluster 1** | Biological Process (GO) | Nucleocytoplasmic transport, Apoptotic process, Mitochondrial translation, mRNA splicing via spliceosome, & DNA repair |
|  | Cellular Component (GO) | Nucleoplasm, Cytosol, Nucleus, Membrane, & Mitochondrion |
|  | Molecular Function (GO) | Protein binding, RNA binding, RNA polymerase II sequence-specific DNA binding transcription factor binding, ATP binding, & DNA binding |
|  | Pathway (KEGG) | Nucleocytoplasmic transport, Th1 and Th2 cell differentiation, Spliceosome, Human T-cell leukemia virus 1 infection, & Th17 cell differentiation |
| **Cluster 2** | Biological Process (GO) | Ubiquitin-dependent protein catabolic process, Protein transport, mRNA splicing via spliceosome, Protein polyubiquitination, & Macroautophagy |
|  | Cellular Component (GO) | Nucleoplasm, Cytosol, Nucleus, Cytoplasm, & Membrane |
|  | Molecular Function (GO) | Protein binding, RNA binding, Ubiquitin protein ligase binding, Ubiquitin-protein transferase activity, & Ubiquitin binding |
|  | Pathway (KEGG) | Amyotrophic lateral sclerosis, Autophagy – animal, Salmonella infection, Pathways of neurodegeneration - multiple diseases, & Non-alcoholic fatty liver disease |
| **Cluster 3** | Biological Process (GO) | - |
|  | Cellular Component (GO) | Cytosol, Nucleoplasm, Golgi membrane, Membrane, & Golgi apparatus |
|  | Molecular Function (GO) | Protein binding, Ubiquitin protein ligase binding, & RNA binding |
|  | Pathway (KEGG) | Adrenergic signaling in cardiomyocytes, mTOR signaling pathway, Proteoglycans in cancer, Coronavirus disease - COVID-19, & MAPK signaling pathway |
| **Cluster 4** | Biological Process (GO) | Cytoplasmic translation, Xenobiotic metabolic process, Estrogen metabolic process, Positive regulation of tyrosine phosphorylation of STAT protein, & Positive regulation of chemokine production |
|  | Cellular Component (GO) | Extracellular space, Extracellular region, Cell surface, Extracellular exosome, & Cytosolic ribosome |
|  | Molecular Function (GO) | Receptor binding, Iron ion binding, Monooxygenase activity, Oxygen binding, & Heme binding |
|  | Pathway (KEGG) | Metabolism of xenobiotics by cytochrome P450, Retinol metabolism, Chemical carcinogenesis - DNA adducts, Drug metabolism - cytochrome P450, & Arachidonic acid metabolism |
| **Cluster 5** | Biological Process (GO) | Cell division, DNA replication, DNA repair, Chromosome segregation, Mitotic sister chromatid segregation |
|  | Cellular Component (GO) | Nucleoplasm, Cytosol, Nucleus, Mitochondrion, & Kinetochore |
|  | Molecular Function (GO) | Protein binding, RNA binding, ATP binding, ATPase activity, & Single-stranded DNA-dependent ATP-dependent DNA helicase activity |
|  | Pathway (KEGG) | Cell cycle, DNA replication, Human T-cell leukemia virus 1 infection, Purine metabolism, & Fanconi anemia pathway |
| **Cluster 6** | Biological Process (GO) | Translation, Positive regulation of peptidyl-tyrosine phosphorylation, Positive regulation of transcription from RNA polymerase II promoter, rRNA processing, & Positive regulation of cell proliferation |
|  | Cellular Component (GO) | Nucleoplasm, Cytosol, Mitochondrion, Membrane, & Cytoplasm |
|  | Molecular Function (GO) | Protein binding, RNA binding, Structural constituent of ribosome, Protein kinase binding, & ATP binding |
|  | Pathway (KEGG) | Ribosome, Pathways of neurodegeneration - multiple diseases, Alzheimer disease, Huntington disease, & Parkinson disease |
| **Cluster 7** | Biological Process (GO) | Extracellular matrix organization, Signal transduction, Cell surface receptor signaling pathway, Regulation of ion transmembrane transport, & Adenylate cyclase-modulating G-protein coupled receptor signaling pathway |
|  | Cellular Component (GO) | Extracellular region, Extracellular space, Plasma membrane, Integral component of plasma membrane, & Cell surface |
|  | Molecular Function (GO) | Receptor binding, Heparin binding, Metalloendopeptidase activity, Extracellular matrix structural constituent, & Cytokine activity |
|  | Pathway (KEGG) | Neuroactive ligand-receptor interaction, ECM-receptor interaction, Protein digestion and absorption, PI3K-Akt signaling pathway, & Renin secretion |
| **Cluster 8** | Biological Process (GO) | Signal transduction, Inflammatory response, Skeletal muscle contraction, Macromolecular complex assembly, & Positive regulation of gene expression |
|  | Cellular Component (GO) | Cytosol, Plasma membrane, Cytoplasm, Membrane raft, & Z disc |
|  | Molecular Function (GO) | Protein binding, ATP binding, Identical protein binding, RNA polymerase II sequence-specific DNA binding transcription factor binding, & Actin binding |
|  | Pathway (KEGG) | MAPK signaling pathway, Lipid and atherosclerosis, Nicotinate and nicotinamide metabolism, NF-kappa B signaling pathway, & Epstein-Barr virus infection |

**Supplementary Table 5.** Drug-target interaction pairs and IC_50_ values.

| **Drugs** | **Target Name** | **Gene Symbol** | **IC_50_ (nM)** | **Cell Line** |
| --- | --- | --- | --- | --- |
| **Benzbromarone** | Cytochrome P450 2C9 | CYP2C9 | 41.00 | *Cebus apella*, & HRPTEpiC cells |
|  | Aldo-keto reductase family 1 member C1 | AKR1C1 | 48.00 | BAOEC cells |
|  | Solute carrier family 22 member 6 | SLC22A6 | 4600.00 | *Drosophila* S2 cells |
|  | Multidrug resistance-associated protein 4 | ABCC4 | 17000.00 | Sf21 cells |
|  | Cytochrome P450 2C19 | CYP2C19 | 18200.00 | Kinetics study |
|  | Cytochrome P450 3A4 | CYP3A4 | 29200.00 | Kinetics study |
|  | Cytochrome P450 1A2 | CYP1A2 | 33100.00 | Kinetics study |
|  | Canalicular multispecific organic anion transporter 2 | ABCC3 | 133000.00 | Sf21 cells |
| **Cimetidine** | Histamine H2 receptor | HRH2 | 500.00 | U2OS cells |
|  | Solute carrier family 22 member 1 | SLC22A1 | 149000.00 | HEK293 cells |
| **Bromoethylamine** | Neuropeptide Y receptor type 1 | NPY1R | 0.06 | SK-N-MC cells |
|  | Mu-type opioid receptor | OPRM1 | 2.66 | CHO cells |
|  | Angiotensin-converting enzyme | ACE | 4.10 | MCF-7, MCF-10 cells |
|  | Protein-glutamine gamma-glutamyltransferase 2 | TGM2 | 6.29 | NIH3T3 cells |
|  | Melatonin receptor | MTNR | 9.98 | HUVEC, & ovine pars tuberalis cells |
|  | G protein-coupled receptor kinase 5 | GRK5 | 11.80 | In-silico study (3D-QSAR) |
|  | Matrix metalloproteinase-9 | MMP9 | 26.00 | Kinetics study |
|  | Glucosamine fructose-6-phosphate aminotransferase 1 | GFPT1 | 150.00 | COS cells |
|  | Anoctamin-1 | ANO1 | 156.50 | FRT, & U251 cells |
|  | Protein Farnesyltransferase chain B | FNTB | 180.00 | NIH3T3 cells |
|  | Delta-type opioid receptor | OPRD1 | 362.00 | CHO cells |
|  | Tyrosine-protein kinase JAK3 | JAK3 | 364.00 | CTLL-2 cells |
|  | DNA repair protein RAD51 homolog 1 | RAD51 | 370.00 | HEK293 cells |
|  | ATP-binding cassette sub-family G member 2 | ABCG2 | 527.50 | H460/MX20 cells |
|  | Aralkylamine N-acetyltransferase | AANAT | 690.00 | Kinetics study |
|  | Gamma-butyrobetaine dioxygenase | BBOX1 | 1200.00 | Kinetics study |
|  | Thymidylate synthase | TYMS | 1288.00 | In-silico study (3D-QSAR) |
|  | Cyclooxygenase | COX | 2000.00 | RAW 264.7 macrophages |
|  | Adenosine receptor A1 | ADORA1 | 4000.00 | DDT1, & PC-12 cells |
|  | Dihydrofolate reductase | DHFR | 5010.00 | HL-60, Bel-7402, BGC823, KB, Hela, & SK-OV-3 cells |
|  | Cytochrome P450 3A4 | CYP3A4 | 13000.00 | Kinetics study |
|  | Telomerase reverse transcriptase | TERT | 15000.00 | HeLa cells |
|  | Histone deacetylase | HDAC | 17000.00 | HeLa cells |
|  | Cyclin-Dependent Kinase 1 | CDK1 | 21500.00 | Indochina clone W2 |
|  | Methionine synthase | MTR | 24715.00 | HL60 cells |
|  | CDK2/Cyclin A/Cyclin A1 | CDK2 | 28500.00 | Indochina clone W2 |
|  | Cyclin-Dependent Kinase 7 | CDK7 | 33400.00 | Indochina clone W2 |
|  | CDK6/cyclin D1 | CDK6 | 34900.00 | Indochina clone W2 |
|  | Cholinesterases | BCHE | 78800.00 | Kinetics study |
|  | Acetylcholinesterase | ACHE | 89100.00 | Kinetics study |
|  | Serum albumin | ALB | 300000.00 | Kinetics study |
|  | Leukocyte elastase | ELANE | 1390000.00 | Kinetics study |
|  | NAD-Dependent Deacetylase Sirtuin-1 | SIRT1 | 1767000.00 | Kinetics study |
|  | Glycogen synthase kinase-3 beta | GSK3B | >100000 | Kinetics study |
|  | Cathepsin K | CTSK | >30000 | Kinetics study |
|  | Cytochrome P450 2C9 | CYP2C9 | >50000 | Kinetics study |
|  | Epoxide hydrolase 1 | EPHX1 | >50000 | Kinetics study |

Part B:

The Akaike information criterion (AIC) and Bayesian information criterion (BIC) are commonly used for probabilistic model selection. Although both methods could perform a similar task, the BIC method has a greater penalty term than AIC. BIC is more useful for the model section where the sample size is much larger than the parameter number (Stoica and Selen, 2004). Since there is a possible overfitting issue when the model has excessive parameter usage, BIC reveals the model performance through consideration of parameter number (*k*), sample number (*n*), and model maximum-likelihood function (*L̂*) (Wit et al., 2012). A model with a low BIC index represents a model that achieves high accuracy with a low number of parameters.

There are three commonly used Log2FC thresholds for screening disease-related genes (positive genes) in differential gene expression analysis: 1, 1.5, and 2. The Log2FC thresholds for the corresponding disease-unrelated genes (negative genes) were set to 0.63, 0.45, and 0.3, respectively, to obtain nearly the same number of disease-unrelated genes. Since there were different Log2FC threshold pairs (positive and negative), the number of samples to be used in the model training would be changed accordingly. Hence, the BIC method was used to find the optimal Log2FC threshold to build the model. When using 0.3 and 2 as the Log2FC thresholds for positive and negative gene lists, the model reached the highest AUC and the lowest BIC value. Therefore, 0.3 and 2 were chosen as the Log2FC threshold for this study.

$\boldsymbol{AIC= -2}\boldsymbol{\cdot ln} \left( \hat{\boldsymbol{L}} \right)\boldsymbol{+2}\boldsymbol{k}$ **(1)**

$\boldsymbol{BIC= -2\cdot}\ln\left( \hat{\boldsymbol{L}} \right)\boldsymbol{+k\cdot}\mathbf{ln}\left( \boldsymbol{n} \right)\boldsymbol{= n\cdot}\ln\left( \frac{\boldsymbol{RSS}}{\boldsymbol{n}} \right)\boldsymbol{+k\cdot}\mathbf{ln}\left( \boldsymbol{n} \right)$ **(2)**

**Table**: Three Different Log2FC Thresholds and the Respective BICs, AUROCs, and Sample Sizes. P: Genes that were associated with LUAD; N: Genes that were not associated with LUAD.

| **Log2FC Threshold** | **AUC** | **BIC** | **Number of Samples** |
| --- | --- | --- | --- |
| P: Log2FC ≥ \|2.0\|  N: Log2FC ≤ \|0.30\| | 0.90 | -1625 | P = 1946, N = 1379 |
| P: Log2FC ≥ \|1.5\|  N: Log2FC ≤ \|0.45\| | 0.84 | -1036 | P = 3462, N =3426 |
| P: Log2FC ≥ \|1.0\|  N: Log2FC ≤ \|0.63\| | 0.75 | -757 | P = 6160, N = 6123 |

**Reference**

STOICA, P. & SELEN, Y. 2004. Model-order selection: a review of information criterion rules. *IEEE Signal Processing Magazine,* 21**,** 36-47.

WIT, E., HEUVEL, E. V. D. & ROMEIJN, J.-W. 2012. ‘All models are wrong...’: an introduction to model uncertainty. *Statistica Neerlandica,* 66**,** 217-236.
